# Nickel oral hyposensitization in patients with systemic nickel allergy syndrome

**DOI:** 10.3109/07853890.2013.861158

**Published:** 2013-11-21

**Authors:** Mario Di Gioacchino, Luisa Ricciardi, Ornella De Pità, Mauro Minelli, Vincenzo Patella, Susanna Voltolini, Valerio Di Rienzo, Marina Braga, Enzo Ballone, Rocco Mangifesta, Domenico Schiavino

**Affiliations:** ^a^ Allergy and Immunotoxicology Unit, Ce.S.I., G. d’Annunzio University Foundation, Chieti, Italy; ^b^ Department of Medicine and Science of Ageing, G. d’Annunzio University, Chieti, Italy; ^c^ Allergology and Clinical Immunology, Messina University, G Martino Hospital, Messina, Italy; ^d^ Immunology and Allergy Unit, Istituto Dermopatico dell’Immacolata IDI-IRCCS, Rome, Italy; ^e^ Departmental Unit of Immunology and Allergology, Internal Medicine, Campi Salentina Hospital, Campi Salentina (Lecce), Italy; ^f^ Allergology and Clinical Immunology, Agropoli Hospital, Agropoli (SA) Italy; ^g^ Allergology Unit, San Martino Hospital, Genoa, Italy; ^h^ Allergology Division, ASL, Frosinone, Italy; ^i^ Allergy Unit, Spedali Civili, Brescia, Italy; ^j^ Department of Allergology, Institute of Internal Medicine, Università Cattolica del Sacro Cuore, Policlinico “A. Gemelli”, Rome, Italy

**Keywords:** Nickel allergy, Allergic contact dermatitis, Systemic nickel allergy syndrome, Systemic contact dermatitis, Nickel-rich food, Nickel oral hyposensitization

## Abstract

*Background:* This is the first randomized, double-blind, placebo-controlled trial (EUDRACT No. 2009-013923-43) evaluating nickel oral hyposensitizing treatment (NiOHT) in patients with “systemic nickel allergy syndrome” (SNAS), characterized by Ni-allergic contact dermatitis and systemic reactions after eating Ni-rich food.

*Methods:* Adults with positive Ni-patch test, who reported symptoms suggesting SNAS, which improved after Ni-poor diet, and were positive to Ni-oral challenge were eligible. Patients were randomly assigned to three treatments (1.5 μg, 0.3 μg, or 30 ng Ni/week) or placebo for a year, with progressive reintroduction of Ni-rich foods form the 5^th^ month. Out of 141 patients randomized, 113 completed the trial. Endpoints were efficacy and tolerability of treatment.

*Results:* During Ni-rich food re-introduction, the 1.5 μg Ni/week group had a mean VAS score significantly higher than placebo (p = 0.044), with significant improvement of gastrointestinal symptoms (p = 0.016;) and significantly fewer rescue medications. Cutaneous manifestations also improved but without reaching statistical significance. After the treatment, oral challenge with higher Ni doses than at baseline were needed to cause symptoms to flare-up in significantly more patients given 1.5 μg Ni/week than placebo (p = 0.05). Patients reported no side-effects.

*Conclusions:* NiOHT is effective in SNAS, in particular on gastrointestinal manifestations, with trend toward improvement of cutaneous symptoms.

Key messages

- Some patients with Ni-allergic contact dermatitis experience skin and gastrointestinal symptoms after eating Ni-rich foods, condition named systemic nickel allergy syndrome.
- Nickel oral hyposensitization allows patients affected by systemic nickel allergy syndrome to safely re-introduce Ni-rich foods, improving their quality of life.

## Introduction

A part of patients suffering from Ni-allergic contact dermatitis (ACD) - which affects 10-20% of the population (1–3) - experiences skin (urticaria/angioedema, flares, itching) and gastrointestinal symptoms (meteorism, colic, diarrhea) after eating Ni-rich foods (4,5). This condition, that according to a recent report (5) affects 20–30% of Ni-ACD patients, is known as systemic contact dermatitis or systemic nickel allergy syndrome (SNAS), the latter better describing the involvement of organs other than skin and the implied immunologic mechanism that involves Th2 as well as ACD's typical Th1 cytokines (6–11).

SNAS patients need to follow a lifelong Ni-poor diet, basically avoiding the majority of vegetables, which poses a potential risk for a deficit in essential elements. To minimize this, nutritionally balanced diets such as BraMa-Ni are now available (5). On the basis of animal studies demonstrating the induction of nickel tolerance after repeated oral doses of metal salts (12,13), the effects of nickel oral hyposensitizing treatment (NiOHT) were studied in humans (open, non-randomized trials), with encouraging results (14–17). Parallel to the clinical improvement, a year of NiOHT significantly reduced *in vitro* nickel-induced Th1 and Th2 cytokines from peripheral blood mononuclear cells compared to baseline. There were no such changes in controls who simply followed a Ni-poor diet for a year (9).

On the basis of these observations, we designed the present trial to study the clinical efficacy and tolerability of NiOHT in SNAS, evaluating the disappearance or reduction of systemic symptoms during the re-introduction of Ni-rich foods, the use of rescue medications and the appearance of side effects.

## Patients and Methods

This was a multicenter, prospective, phase III, randomized, double-blind, placebo-controlled trial with four parallel groups (three treatment doses and placebo) conducted in eight university or hospital allergology units in Italy (Chieti, Agropoli, Genoa, Latina, Lecce, Messina, Rome Catholic University, Rome IDI). Ethics committees in all centers approved the protocol. Enrolled patients gave their signed informed consent.

### Eligibility criteria

Eligible participants were adults who i) had a positive Ni-patch test, ii) reported symptoms suggesting SNAS (5), iii) improved at least 70% from baseline after one month on a Ni-poor diet (severity of symptoms rated on a visual analog scale - VAS), and iv) were positive to a Ni-oral challenge (NOC).

Exclusion criteria were pregnancy and lactation, concomitant treatment with steroids and/or antihistamines, inability to give the informed consent, and participation in another study.

### Interventions

As treatment, patients took hard gelatin capsules containing different doses of Ni- as NiSO_4_·6H_2_O with microcrystalline cellulose as excipient, or placebo, all identical in appearance and flavor. Eligible patients used the BraMa-Ni diet (5) for one month, and were then randomly assigned to four groups. The first three groups were given nickel at doses increasing in 40 days from 1 ng to the three final maintenance doses (10 ng, 0.1 μg and 0.5 μg, corresponding to a dose of NiSO_4_ of 0.044 μg, 0.44 μg and 2.2 μg respectively), three times a week for 12 months. To protect the blinding, patients randomized to lower doses received placebo for the first days of the up-dosing phase, according to the scheme in Table 1.

**Table 1.** Scheme of up dosing and maintenance of nickel oral hyposensitization of the four groups.

Days	Group 1Ni dose	Group 2Ni dose	Group 3Ni dose	Group 4
1–10	1 ng/day	Placebo	Placebo	Placebo
11–20	10 ng/day	1 ng/day	Placebo	Placebo
21–30	0.1 μg/day	10 ng/day	1 ng/day	Placebo
31–40	0.5 μg/day	0.1 μg/day	10 ng/day	Placebo
41–320	0.5 μg 3 times a week	0.1 μg 3 times a week	10 ng 3 times a week	Placebo 3 times a week

All eligible patients after one month of Ni-pour diet were randomly assigned to one of the four groups of treatment. Nickel dose was progressively increased in 40 days from 1 ng to 3 definite maintenance doses (10 ng, 0.1 μg and 0.5 μg) administered 3 times a week for a total of 12 months. In order to protect the blinding, patients randomized to lower doses received placebo during the first days of the up-dosing phase.

Nickel oral challenge was done by administering weekly increasing doses of nickel, from 1.25 to 6 μg (with increment of 1.25 μg), until the appearance of clinical manifestations of SNAS, in particular taking into account of objective data as the appearance of cutaneous (urticaria, angioedema, eczema, erythema) or gastrointestinal (evident meteorism, diarrhea, vomiting, colic). Capsules were made by Lofarma SpA, Milan, Italy.

All patients were allowed rescue medications (desloratadine 5 mg/day and/or prednisone 25 mg/day) if necessary.

### Outcomes

Primary endpoints: 1) disappearance or reduction of symptoms during Ni-rich food re-introduction (patients reported their clinical status in a diary and made a VAS at each control visit), and use of rescue medications (reported in the diary); 2) tolerability evaluated on the basis of side effects (reported in the diary).

Ancillary analyses: in a substantial number of patients, patch tests became negative after the treatment and the NOC doses able to elicit symptoms at the end of the study changed from baseline. Therefore, since the patch test and NOC were included in the protocol, these findings were analyzed, even though this was not pre-specified.

### Study protocol

Time 0 (T0)/Screening: history, clinical evaluation, laboratory tests, pregnancy test, patch test, VAS, prescription of 30-day BraMa-Ni diet to eligible subjects.

T1 visit (end of diet): Clinical evaluation, VAS, and NOC in patients whose VAS rating improved at least 70% from baseline. NOC-positive patients were randomized for treatment and received Ni capsules, diary and rescue medications.

T2 visit (3rd month of treatment): clinical and diary evaluation, VAS. Prescription to re-introduce foods with maximum 100 μg/kg nickel content during the 5th month, then up to 200 μg/kg during the 6th month of treatment (Table 2).

**Table 2.** Foods by nickel content.

**Ni 100 μg/Kg**	**Ni 200 μg/Kg**	**Ni 500 μg/Kg**	**Ni > 500 μg/Kg**
Carrots Figs Lettuce Green Salad Licorice Mushrooms Plaice and Cod Rhubarb Rice Tea	Apricots Broccoli Corn Eggplant Lobster Onions Peppers Pears Raisins Zucchini	Artichoke Asparagus Beans Cabbage Cauliflower Green Beans Integral Flour Yeast Margarine Mussels Oysters Potatoes Peas Plums Spinach Tomatoes	Almonds Chickpeas Cocoa and Derivatives Concentrated Tomato Lentils Oats Peanuts Walnuts

The reintroduction of Ni-rich foods started from the 5^th^ month with foods with maximum 100μg/Kg nickel content, until 200μg/Kg during the 6^th^ month, until 500 μg/Kg during the 7^th^ month and then all other Ni-rich foods from the 8^th^ month.

List of Ni content in foods has been derived from literature (26–29).

T3 visit (7th month of treatment): clinical and diary evaluation, VAS. Prescription to re-introduce foods with maximum 500 μg/kg nickel content for a month, then all other Ni-rich foods (Table 2).

T4 visit (end of treatment): clinical and diary evaluation, VAS, patch test, NOC, laboratory tests.

Unscheduled visits were allowed at patients’ request and clinical data were recorded.

### Visual Analogic Scale

Patients were asked to show in a 10 cm Visual Analogic Scale their perceived clinical condition (with 0 the worst and 10 the best) at enrollment and at each control visit.

### Sample size/power/level of significance

Published studies report that NiOHT was effective (disappearance of symptoms on re-introduction of Ni-rich foods) in 60% of SNAS patients, as opposed to 30% disappearance of symptoms in the placebo or untreated groups. Setting the type I error at 5% (α = 0.05) in a one-tailed test of significance and a type II error of 20%, which corresponds to a study power of 80%, the calculated sample size indicated 30 patients for each of four treatment arms. Considering a drop-out rate of 30–40%, a total of 160 patients were enrolled and 40 were randomly assigned per treatment arm. PASS 2005 software (Kaysville, UT) was used.

### Randomization

We used a computer-generated list of random numbers to allocate participants, with the randomization sequence stratified by center with 1:1:1:1 allocation. Capsules were pre-packed in blister packs and consecutively numbered for each patient according to the randomization schedule. Each patient was assigned an order number and received the corresponding packs. The contract research organization that controlled the quality standard throughout the trial prepared the allocation sequence and stored the allocation list. Investigators enrolled patients and assigned them to their specific groups.

### Statistical analysis

Patients who reached constant dosage were included in the intention-to-treat (ITT) analysis. Categorical variables were summarized as frequency and percentages. Continuous variables were presented as mean and standard deviation (SD). Efficacy was defined as the proportion of patients in each of the four treatment arms whose symptoms were either reduced or eliminated. The chi-square test with Yates’s continuity correction or Fisher's exact test was employed to examine differences in efficacy. Intergroup differences in continuous variables were analyzed by ANOVA and the t-test. The confidence interval (95% CI) was calculated for the mean difference and percentage risk difference between groups. The equivalent non-parametric tests were used when the data were not normally distributed or the sample was too small. All hypotheses were tested considering a single tail. P values of 0.05 or less were considered statistically significant. SPSS^®^ Advanced Statistical^™^ 13 (2004, Chicago, IL) was used.

## Results

**Figure 1.**
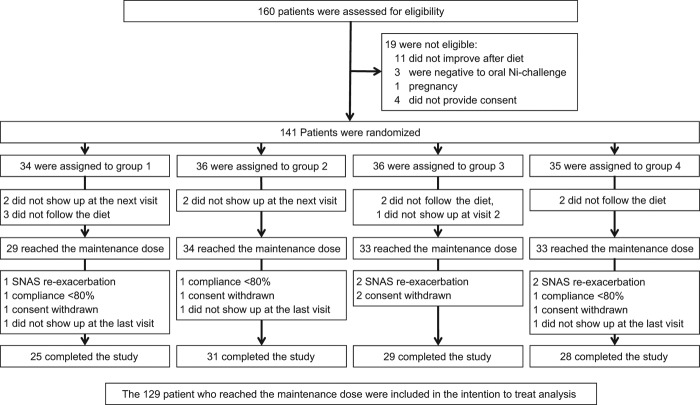
Enrolment, randomization and follow-up of the study.

Recruitment started in April 2010 and ended in May 2012. Figure 1 illustrates the flow diagram of the trial. Out of 141 patients randomized, 129, who reached the constant dosage of NiOHT, were included in the intention-to-treat (ITT) analysis. 113 of them completed the trial.

### Symptoms

The main demographic characteristics of enrolled patients and their symptom severity at enrolment (rated on the VAS) are summarized in Table 3, where symptoms reported by 129 patients included in the ITT analysis are also reported. As per inclusion criteria, NOC, performed after one month of BraMa-Ni diet, elicited the reappearance of SNAS symptoms (cutaneous and gastrointestinal in particular, but not cough and headache).

**Table 3.** Main baseline characteristics of patients in the four study groups.

Variables	Group 1 (34)	Group 2 (36)	Group 3 (36)	Group 4 (35)	p-value
Sex, no. (%)					0.627^¥^
Male	2 (5.9)	1 (2.8)	1 (2.8)	3 (8.6)	
Female	32 (94.1)	35 (97.2)	35 (97.2)	32 (91.4)	
Age, yrs*	27.6 ± 10.2	36.4 ± 10.2	38.7 ± 9.7	40.3 ± 9.8	0.415^†^
Weight, kg*	61.8 ± 9.2	61.3 ± 9.0	60.4 ± 10.3	66.1 ± 17.2	0.201^†^
Height, cm*	163.1 ± 6.2	163.7 ± 6.7	161.1 ± 5.8	163.9 ± 8.0	0.263^†^
BMI*	23.2 ± 3.1	22.9 ± 3.5	23.4 ± 4.5	24.6 ± 6.4	0.436^†^
VAS, T0 *median (range)*	1.9 (1.0–5.0)	2.2 (1.0–6.0)	3.0 (1.0–8.0)	2.0 (1.0–6.4)	0.233^¥^
VAS, T1 *median (range)*	8.0 (4.3–9.9)	8.0 (4.3–10.0)	8.0 (5.0–9.0)	8.1 (3.5–9.7)	0.575^¥^
Skin symptoms (ITT pts) T0/NOC	29/29	34/34	33/33	33/33	
GUT symptoms (ITT pts) T0/NOC	28/28	32/32	32/32	31/31	
Cough (ITT pts) T0/NOC	2/0	0/0	3/0	1/0	
Headache (ITT pts) T0/NOC	5/0	4/0	4/0	2/0	

BMI: Body-mass index (weight in kilograms divided by the square of the height in meters); NOC: Nickel Oral Challenge; ITT: Intention to treat analysis.

There were no significant differences between the four groups in demographic characteristics or severity of symptoms, rated on a visual analog scale (VAS) at enrollment and after one month of Ni-poor diet.

Distribution of symptoms in the four groups at enrollment and after NOC has been reported for patients included in the ITT analysis.

Skin symptoms comprehend, associated or not, the following: urticaria, angioedema, eczema in region without direct contact with nickel.

GUT symptoms comprehend, associated or not, the following: meteorism, gastric acidity, abdominal colic, diarrhea, vomit, acidity to the throat.

*Data are mean ± SD. ^†^ANOVA; ^¥^Kruskal-Wallis test for independent samples.

As expected, considering the inclusion criteria, all clinical parameters of all groups had significantly improved from baseline at T1 (after the first month of diet), with no significant differences between the groups (non-parametric tests, p < 0.05) (Table 3).

### Outcomes

There were no significant differences in VAS and the various symptoms among the groups at the T2 and T3 visits. At T4, with the re-introduction of the highest Ni-containing foods (500 μg/kg and over), group 1 (given the highest Ni dose) showed the best values for all parameters, groups 3 and 4 the worst and group 2 intermediate. Only for group 1 were the changes from baseline significantly better than placebo; the significance was amplified when considering groups 3 and 4 together as placebo. There were no differences between groups 3 and 4 in any parameter, and in some cases group 3 patients were worse than placebo.

### VAS ratings

**Figure 2.**
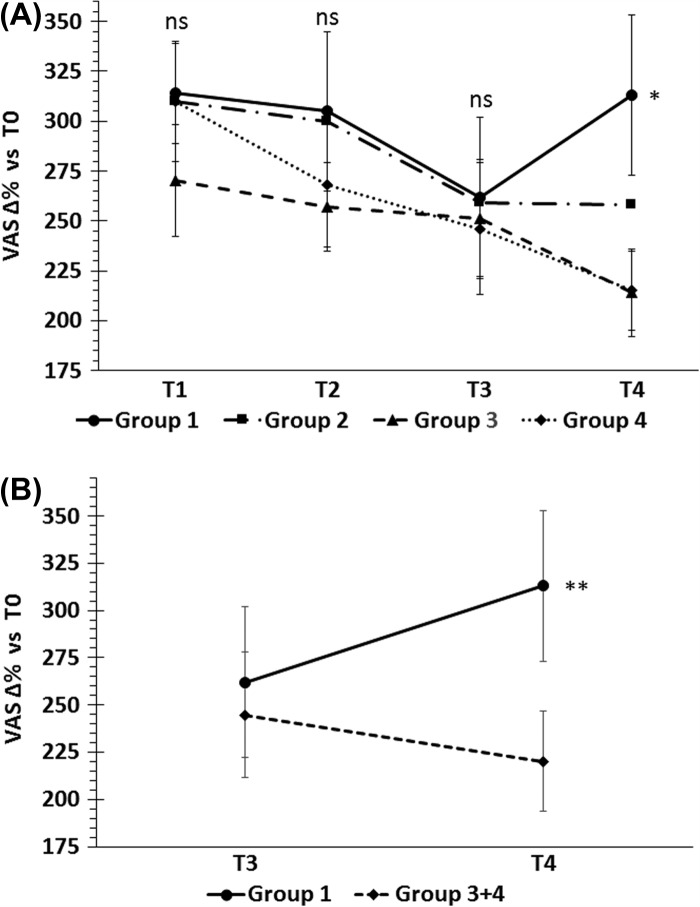
Percent changes of VAS from baseline during the study. (A) No significant differences were found at T1, T2 and T3 visits, whereas at T4, when Ni-rich foods were re-introduced, the mean VAS score of group 1 (receiving the highest Ni dose) was significantly higher than the placebo group *p < 0.048, t-test. (B) Combining group 3 and group 4 raised this significance (groups 3 and 4 had similar results and group 3 dose can be considered a placebo).*p < 0.038, t-test.

Figure 2 shows the changes in VAS scores from baseline. The mean score for group 1 was significantly higher than group 4 (t-test p = 0.048; mean difference 98.92; 95% CI 0.99 to 196.83) and groups 3 and 4 together (t-test, 0.038; mean difference 98.76; 95% CI 5.40 to 192.12). At T4, the mean VAS score for group 1 was similar (+ 2.3%) to that at T1 (after the Ni-poor diet), while that of group 4 decreased 26.9%.

### Gastrointestinal and cutaneous symptoms, headache and cough

Changes in gastrointestinal and cutaneous symptoms paralleled the VAS scores. However, while gastrointestinal symptoms in group 1 at T4 showed significantly more improvement than the placebo group (group 1 vs. 4 – Fisher's exact test p = 0.016; risk difference 26.6; 95% CI 5.1 to 46.1) (Table 4), improvements in cutaneous manifestations were not significant, p = 0.065. In all, 20 patients in group 1 (69%) reported complete disappearance of skin symptoms at T4, compared to 12 (35.3%) in Group 4 and 13 (39.4%) in Group 3.

**Table 4.** Changes in gastrointestinal symptoms in group 1 compared to placebo at T4.

Endpoint	Group 1 (ITT)	Group 2 (ITT)	Group 3 (ITT)	Group 4 (plb) (ITT)
*GI symptoms:* Improved Not improved	(29)* 23 (79.3%) 1 (3.4%)	*(34)* 25 (73.5%) 7 (20.5%)	(33)* 16 (48.5%) 13 (39.4%)	(33)* 18 (54.5%) 8 (24.2%)

There were significant differences between groups 1 vs 3 and group 1 vs 4 at T4, during the re-introduction of Ni-rich foods.

ITT: Intention to treat analysis.

*Fisher’s exact test = 0.016; Risk difference (95% CI): 26.6 (5.1 to 46.1).

Headache and cough did not significantly change during the study, and no significant differences were found between groups (data not shown).

### Rescue medications

At T4, with the re-introduction of Ni-rich foods, three patients in group 1 took rescue medications compared with 17 in group 2, 12 in group 3 and 11 in group 4 (each group vs. group 1, Fisher's exact test, p < 0.05).

### Ancillary analyses

**Figure 3.**
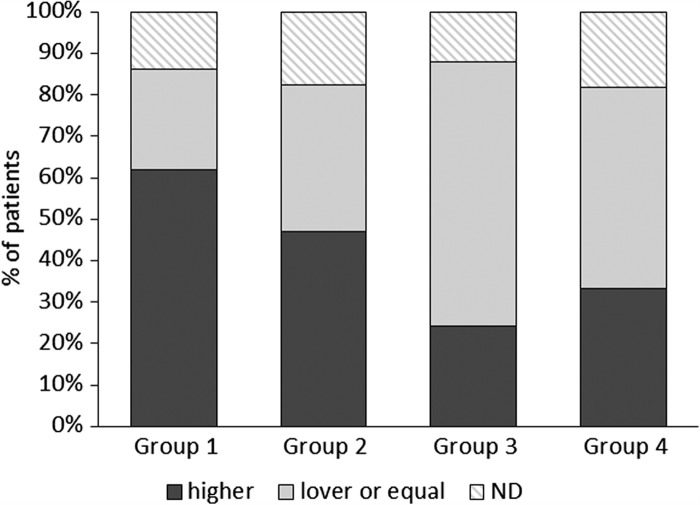
Percent of patients (respect to those included in the intention to treat analysis) with higher or lower/equal NOC doses at the end of the study respect to T1. The nickel oral challenge (NOC) was compared on the basis of the nickel dose required to induce flare-up of SNAS symptoms. After the treatment, oral challenge with higher Ni doses than at baseline were needed to cause symptoms to flare-up in significantly more patients given 1.5 μg Ni/week than placebo: Risk difference (95% CI): 27.6 (0.8 to 49.3); p = 0.05. Comparing Group 1 to Group 3 + 4 the significance increased: Risk difference (95% CI):38.1 (14.5 to 55.7); p = 0.002.

**Figure 4.**
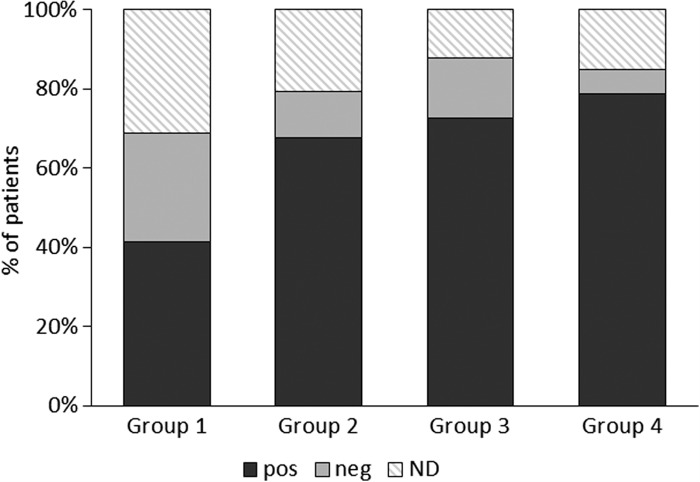
Positive and negative patch tests in the four groups at the end of the study; as per inclusion criteria, all patients were patch test-positive at baseline. Values are expressed as percentage of patients evaluated in the intention to treat analysis. There were significantly more patch test-negatives in group 1 (Risk difference (95% CI): 32.9 (9.0 to 54.8); p = 0.008). Pos: positive patch test; Neg: negative patch tests; ND: not done.

At the end of the study, a significantly larger number of group 1 patients than at baseline needed a NOC with higher Ni doses to induce a flare-up of symptoms compared to placebo (Fisher's exact test p = 0.05; risk difference 27.6; 95% CI, 0.*8* to 49.3) or placebo plus group 3 (Fisher's exact test p = 0.002; risk difference 38.1; 95% CI 14.5 to 55.7) (Figure 3). Significantly more patients had a negative patch test at the end of the study in group 1 than group 4 (Fisher's exact test p = 0.008; risk difference 32.9; 95% CI 9.0 to 54.8) and group 3 + 4 (Fisher's exact test p = 0.011; risk difference 27.7; 95% CI 0.8 to 49.3) (Figure 4).

### Side effects

Only one patient in group 1 reported gastrointestinal symptoms after the 10th dose of 0.5 μg Ni. The patient took desloratadine and symptoms disappeared within 3 hours. The patient concluded the study as scheduled.

## Discussion

This is the first double-blind, randomized, placebo-controlled trial evaluating the efficacy of NiOHT in patients with SNAS. The treatment was effective. During the re-introduction of Ni-rich foods, symptoms improved significantly in patients given the highest Ni dose, compared to placebo, with a VAS score similar to that of patients in Ni-pour diet. The effect of NiOHT seemed dose-dependent, as 1.5 μg Ni/week gave the best results, 30 ng Ni/week and placebo the worst and 0.3 μg Ni/week was intermediate. Gastrointestinal symptoms significantly improved, parallel with VAS scores, compared to placebo (Table 4 and Figure 2), and were more sensitive to NiOHT than cutaneous manifestations, which decreased in frequency, but without reaching statistical significance (p > 0.05) compared to group 3 and placebo. This is not altogether surprising, as the skin contact with nickel, which can never be completely avoided, might have induced symptoms linked to ACD, confounding the results. Cough and headache, reported by patients as linked to the ingestion of Ni-rich foods, did not significantly change during the study, nor were induced by NOC. A recent publication (5) confirmed that they seemed not to be part of the syndrome as they were never induced by NOC, and did not reappear with the re-introduction of Ni-rich foods after amelioration induced by a nickel low diet.

The effectiveness of NiOHT with 1.5 μg Ni/week is corroborated by the observation that during the re-introduction of Ni-rich foods only three patients (10.3%) took rescue medications, compared to significantly more in other groups (group 1 vs. each group, p < 0.05). The subjective data, symptoms and VAS ratings, which show post-NiOHT tolerance to nickel, are supported by objective tests such as NOC and patch test. At the end of treatment, significantly more patients in group 1 than in group 3 and the placebo group needed a higher nickel dose at the oral challenge to elicit symptoms than before treatment. Similar significant differences were found between group 1 and groups 4 or 4 + 3 for patients with a negative patch test at the end of the study.

No comparison is possible with other trials, as all previous studies on the effects of NiOHT were unregistered and open (14–17), only one randomized trial comparing patients treated with active medications and controls receiving only a Ni-poor diet (9). Results were positive, as only the treated patients were able to re-introduce the majority of Ni-rich foods. Other trials in patients with ACD but no systemic symptoms gave contrasting results. Open studies (18–20) were positive, while the double-blind one indicated that NiOHT reduced the T-cell *in vitro* response to nickel but failed to improve the clinical expression of ACD (21). In addition, the various trials on SNAS are hard to compare because of the wide range of nickel doses (from 0.1 ng to 0.5 μg), frequency of dosing (daily to weekly) and diagnostic methods (essentially based on history, though a few on specific NOC). One open randomized trial found that, parallel with clinical efficacy, lymphocytes from NiOHT-treated patients released significantly less nickel-induced IFN-γ, IL-13 and IL-5 *in vitro* than at baseline, whereas there were no significant changes in Ni-poor diet controls (9).

The involvement of Th2 cytokines in the pathogenesis of SNAS has been reported in various studies (10,11), showing a significant dose-dependent increase of serum IL-5 in SNAS, but not in ACD patients. Significant decreases of CD3 + CD45RO+ CLA+ and CD8 + CD45RO+ CLA+ blood lymphocytes have also been seen, with massive infiltration of CD4 + cells in the duodenal lamina propria and epithelium (to our knowledge the only pathology with CD4 + cell infiltration in the epithelium) (7). A study on the immunomodulatory properties of NiOHT also showed an increase of IL-10 (17), a regulatory cytokine involved in the action of vaccines for inhalant and hymenoptera venom allergy (22). The cytokine changes, first of all of regulatory cytokines, led to the hypothesis that nickel tolerance after NiOHT might be a consequence of the differentiation and proliferation of nickel-specific T regulatory lymphocytes, which can maintain immune tolerance to Ni in healthy subjects (23). This also can explain the effect of the low Ni doses administered. In fact, high doses of antigen favor an anergy-driven pathway to tolerance while low doses of antigen promote a suppressive pathway via regulatory T cells producing IL-10 and TGF-β (24).

Clinical aspects of SNAS was described by Braga et al (5). The most frequent manifestation of SNAS was the flare-up of previous ACD eczematous lesions reported by all patients, followed by a flare-up of a previously positive nickel patch test. Such symptoms were variably associated with eczema in regions not in contact with the metal and/or with urticaria and angioedema. In almost all cases patients reported meteorism and dyspepsia combined with colic, gastric acidity, vomit, diarrhea or throat acidity; less than 10% of patients experienced gut symptoms without skin manifestations. Lactose intolerance was found in a high percentage of SNAS patients.

We think that SNAS is closely related to the systemic contact dermatitis (SCD), were only cutaneous symptoms are considered, but in our experience almost all patients previously diagnosed as SCD to nickel have gastrointestinal symptoms. Many other metals are considered to induce SCD, such as cobalt, chromium, and zinc that are ubiquitous in our environment, with cutaneous manifestations like urticaria/angioedema, lichen planus, palmoplantar pustulosis, and maculopapular rash. Also in these cases, the diagnosis of sensitivity to metal is established by epicutaneous patch testing and oral metal challenge (25). In these cases, contrary to Nickel sensitivity, we never found gastrointestinal symptoms.

Limitations of the present study are related to the determination of nickel doses, sample size, duration of the treatment. Nickel doses during the trial were established in relation to those used in the majority of studies, but the lowest gave exactly the same results as placebo, and we do not know whether doses higher than the largest we used would have given better results or might have caused side effects. The difficulty in determining Ni doses for treatment is also underlined by the fact that Ni given with NiOHT is immediately bioavailable, on the contrary little is known about Ni speciation and bioavailability of Ni introduced with foods. Furthermore, the lack of published data made it hard to select the best parameters for adequate sample size. The number of patients enrolled seems to have been too low, and in fact, on combining group 3 (patients receiving the lowest nickel dose) with the placebo group, the statistical significance appeared clearer. In a larger sample, we might have been able to gain a better picture of NiOHT's effects on skin symptoms. Similarly, we do not know if one year is long enough to obtain stable results, so this needs to be verified by an adequate period of follow-up. Furthermore, we did not perform placebo controlled NOC, anyway being the study blinded and randomized the possible biases were distributed among groups and do not affect the overall result of the trial.

In conclusion, the results of the study have substantial clinical implications, considering the large numbers of patients with SNAS (in a recent study rated as 20–30% of all Ni-ACD patients), whose quality of life is diminished on account of their lifelong dietary restrictions. NiOHT allows patients to re-introduce Ni-rich foods in the absence of substantial side effects.

## Trial Registration

The trial was registered in EUDRACT with No. 2009–013923-43
